# Effect of Maillard reaction conditions on the solubility and molecular properties of wheat gluten–maltose conjugates

**DOI:** 10.1002/fsn3.1869

**Published:** 2020-09-17

**Authors:** Yongling Song, Shaoming Yang, Jianghe Li

**Affiliations:** ^1^ Henan Key Laboratory of Cereal Resource Transformation and Utilization School of Food Science and Technology Henan University of Technology Zhengzhou China

**Keywords:** FTIR spectroscopy, gluten, Maillard reaction, maltose, solubility

## Abstract

In this experiment, the conjugation reaction between gluten and maltose via Maillard reaction under dry‐heated condition was studied. The process conditions for the preparation of protein–maltose conjugates with optimum solubility were optimized by using Box‐Behnken model. The conjugation reaction and the structure changes of the protein–maltose conjugates were confirmed by infrared spectroscopy (FTIR) and scanning electron microscopy (*SEM*). The results showed that the process conditions for the preparation of protein–maltose conjugates with optimum solubility were as follows: temperature 50.72°C, time 1.92 days, and gluten/maltose (W/W) 267.36%. The infrared spectroscopy showed that the structure of the modified protein had a very obvious change, including the decrease in β‐fold and β‐turn and the increase in α‐helix at a certain degree. But the conjugation reaction has little effect on the irregular coiled structure. The scanning electron microscopy showed that the microstructure of gluten is small grainy, but gluten–maltose conjugate looks sheet with bigger volume.

## Graphical abstract

In this experiment, gluten was modified through by glycosylation. The solubility of modified gluten was improved. The structure of modified gluten has changed that confirmed by infrared spectroscopy and scanning electron microscopy.

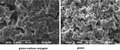

## 1 INTRODUCTION

Gluten is a powdered product made from wheat that is washed with water to remove starch and other water‐soluble substances (Gottardi, Hong, Ndagijimana, & Betti, 2014). It accounts for about 10% of the weight of wheat grain. Its protein content is up to 75%–85%, containing 15 essential amino acids, and it is a nutritious and cheap plant protein source (Amiri, Farshi‐Marandi, & Shahedi, 2019). As a functional food ingredient, food improver, and food additive, gluten has the characteristics of large quantity, low price, high protein content, good flavor, complete amino acid composition, etc. It plays an important role in flour products, meat products, dairy products, cold drink products, powder oil, and other foods (Mahroug et al., 2020). Because there are a lot of nonpolar amino acid residues such as proline and leucine in the molecular structure of wheat gluten protein and nondissociable polar glutamine residues, gluten protein mainly exists in large aggregates under neutral conditions. So there are many limitations in the functional characteristics of wheat gluten protein, especially its solubility and emulsification, which are difficult to meet the requirements of food industry. It greatly limits the application of gluten (Hwang et al., 2017). Therefore, improving the solubility of wheat gluten is of great significance to broaden its scope of application.

In order to improve the functional characteristics of protein, many studies have been made such as chemical, physical, or enzyme treatments (Apichartsrangkoon, 2003; Asrarkulova & Bulushova, 2018; Klompong, Benjakul, Kantachote, & Shahidi, 2007; Lawal, Adebowale, & Adebowale, 2007; Wang et al., 2008). During these conventional modification methods, the physical modification often depends on the mechanical strength, such as high pressure or shear (Galazka, Dickinson, & Ledward, 2000; Haykawa, Linko, & Linko, 1996). Due to the appearance of the potential health hazards or dangerous products, most of the chemical modifications are not used in the food industry. The enzymatic treatments often lead to bitter taste, affecting the food's flavor. Therefore, more appropriate method needs to be applied to protein modification.

Protein and saccharide are two types of biological macromolecules in the food system, the main factors influencing food function and texture. From the beginning of the 90 s, several research groups in Europe and Japan began to covalent complexes of protein–saccharide. The ε‐lysyl amino groups of protein and the reductive terminal carbonyl group of carbohydrate were linked by Amadori‐type linkage to form protein–saccharide complex (Kato, Minaki, & Kobayashi, 1993). The functional properties of protein–saccharide conjugates are mainly based on the protein. The introduction of saccharide is modified or enhanced functions for proteins. At present, there are many studies using different sugars for protein modification, including monosaccharides and polysaccharides (Hiller & Lorenzen, 2010; Sheng et al., 2020). Protein–saccharide Maillard‐type conjugates obtained under controlled conditions have better functional properties than natural proteins, such as thermo stability, emulsifying capacity, and foaming properties (Nasrollahzadeh, Varidi, Koocheki, & Hadizadeh, 2017; Nooshkam, Varidi, & Bashash, 2019; Pirestani, Nasirpour, Keramat, Desobry, & Jasniewski, 2017; Sun et al., 2018; Zha, Dong, Rao, & Chen, 2019; Zhong et al., 2019). And they also have remarkable solubility and stability against pH changes, ionic strengths, high temperature, and shear rates, which make them promising bioactive compounds in the food industries (Nooshkam, Babazadeh, & Jooyandeh, 2018; Xu, Zhao, & Bian, 2018; Xu et al., 2020). In addition, some proteins after glycosylation have antioxidant activity (Nooshkam & Madadlou, 2016) and antiallergic property (Tian, Liu, Zhang, Tao, & Xue, 2020).

The glycosylation of protein due to the mild reaction conditions does not add the foreign chemicals and is generally considered suitable for application in the food industry (Shepherd, Robertson, & Ofman, 2000). Because of the important role of Maillard reaction in food stability, flavor development, nutrition, and health, it is important to develop reasonable methods to reduce the adverse consequences of this reaction and to optimize the beneficial effects, while trying to establish conditions under which the highest yield of carbohydrate binding proteins can be produced (Scaman, Nakai, & Aminlari, 2006). Therefore, the purpose of this study was to evaluate the effects of temperature, time, and the addition ratio of reactants on the degree of glycosylation of gluten and maltose to obtain mixtures with different glycosylation levels and to evaluate their functional properties.

## 2 MATERIALS AND METHODS

### 2.1 Materials

Wheat gluten was obtained from Fengqiu County HuaFeng powder industry Co., Ltd, and contained 73.53% protein, 0.73% fat and 11.14% water content, and 1.04% ash content; maltose was purchased from Shanghai Jingchun biochemical technology Co. Ltd; corn oil was purchased from local markets and used without further refinement; and all other chemicals were of analytical grade.

### 2.2 Preparation of protein–maltose conjugates

Gluten (1.0 g) was dissolved in 100 ml 0.05 mol/L sodium phosphate buffer with pH 12. Maltose powder (5, 3.6, and 3 mg) was added into gluten solution, respectively. The sample solutions were stirred at room temperature with a magnetic stirrer at 150 *g* for 2 hr to dissolve the mixture completely. The solutions were then freeze‐dried. Each experiment included a control sample without maltose. Freeze‐dried powders were reacted at 45, 50, and 55°C at 79% relative humidity (over saturated potassium bromide) for up to 1, 2, and 3 days.

### 2.3 Solubility

The solubility was determined by biuret method with bovine serum albumin as a standard. Native and glycated gluten were diluted in distilled water (1%, W/V). The samples were set to stand for 30 min. And then samples were centrifuged for 10 min at 300 *g* . Protein content in the supernatant was determined. 1 ml of the supernatant (or water as the blank) put into the test tube, and 4 ml of biuret reagent was added. After that, the solution was mixed gently and thoroughly immediately. Incubation was carried out at 25°C for 30 min. Finally, the solution absorbance of each tube was measured at 540 nm by UV–VIS spectrophotometer.The measurement was carried out in triplicate for each sample.

### 2.4 Fourier transform infrared spectroscopy (FTIR)

Fourier transform infrared spectroscopy was determined by using Madhav P. Yadav's method (Yadav, Strahan, Mukhopadhyay, Hotchkiss, & Hicks, 2012). The gluten and graft were thoroughly dried, and then, the samples were mixed with KBr at a mass ratio of 1:300 and then thoroughly ground and pressed into thin slices. An infrared spectrometer was used to perform a full‐band scan (400 ~ 4000 cm^−1^). The sample was scanned for 12 times.

### 2.5 Scanning electron microscopy

Scanning electron microscopy was performed according to the method of Li et al. (2012). Gluten and gluten–maltose conjugates are fully dry, take a small amount of sample affixed to the sample sets of conductive adhesive, spray on the surface of conductive adhesive under vacuum conditions gold, and then scan them using scanning electron microscopy. Digital images of topographical features of the samples were collected using a Hitachi S‐4300 environmental scanning electron microscope under the high vacuum/secondary electron imaging mode at an accelerating voltage of 25 kV and instrumental magnification 500×.

### 2.6 Experimental design

The Box‐Behnken model in software Design Expert 7.1 was used to achieve the optimal condition for Maillard reaction. The independent variables were reaction temperature (X_1_, °C), gluten/ maltose ratio (X_2_, %), and reaction time (X_3_, d), while the dependent variable was solubility (Y, mg/ml). Replicates were performed for each experiment, and the average values were recorded as the response. The experimental Y value was fitted to the following quadratic equation:

$$Y=A_{0}+\sum_{i=1}^{3}A_{i}X_{i}+\sum_{i=1}^{3}A_{\mathrm{ii}}X_{i}^{2}+\sum_{i=1}^{2}\sum_{i=i+1}^{3}A_{\mathrm{ij}}X_{i}X_{j}$$

where *Y* is the dependent variable, *A*_0_ is a constant coefficient, A_i_ is linear coefficient, *A*_ii_ is quadratic coefficient and *A*_ij_ is interaction coefficient between different factors, and *X*_i_ and *X*_j_ are the coded values of the independent variables.

According to the preliminary test results and actual production requirements, the horizontal coding table that identified the three factors is shown in Table 1. After the test according to the test scheme, the regression equation is obtained by quadratic regression fitting of the test data.

**Table 1:** The code table of three factors and three horizontals for Response Surface Methodology

Horizontal	Reaction temperature X_1_/°C	Gluten/maltose ratio (W/W) X_2_/%	Reaction time X_3_/d
−1	45	200	1
0	50	275	2
1	55	350	3

### 2.7 The data analysis

The data in the test were the mean value of three tests, and the software Design Expert 7.1 was used to analyze the regression model.

## 3 RESULTS AND DISCUSSION

### 3.1 Optimization of the Maillard reaction condition

#### 3.1.1 Statistical analysis and the model fitting

The test is carried out according to the experimental design scheme, and the results are shown in Table 2. Using the software Design Expert 7.1 to perform regression analysis of solubility measured under different conditions in Table 2, the regression equation of solubility was obtained by software regression fitting:

**Table 2:** The test result for Response Surface Methodology

Test serial number	X_1_/°C	X_2_/%	X_3_/d	Solubility Y/(mg/ml)
1	1	1	0	0.78
2	1	0	1	0.87
3	1	0	−1	0.89
4	1	−1	0	0.98
5	0	1	1	0.83
6	0	1	−1	0.85
7	0	0	0	1.6
8	0	0	0	1.61
9	0	0	0	1.59
10	0	0	0	1.57
11	0	0	0	1.57
12	0	−1	1	0.83
13	0	−1	−1	1.06
14	−1	1	0	0.62
15	−1	0	1	0.42
16	−1	0	−1	0.66
17	−1	−1	0	0.65

Y_2_ = +1.59 + 0.15 × X_1_ − 0.055 × X_2_ − 0.064 × X_3_ − 0.043 × X_1_×X_2_ + 0.055 × X_1_×X_3_ + 0.053 × X_2_ × X_3_ − 0.51 × X_1_^2^ − 0.32 × X_2_^2^ − 0.37 × X_3_^2^ and the coefficient of determination (*R* ^2^) is .8845. Then, ANOVA was used to analyze the significance and applicability of the experimental model, and statistical summary was provided (Table 3). As seen in Table 3, the *p* value of the model was much <.01, which indicates the significance of the model for Maillard reaction. This shows that the model coincides with the experimental data very well. The linear variables X_1,_ X_2_, and X_3_ showed statistically significant influences (*p* < .01); and the quadratic variables X_1_^2^_,_ X_2_^2^, and X_3_^2^ also significantly influenced the solubility (*p* < .01). The interaction coefficients between the independent variables also showed statistically significant influences (*p* < .05). However, the *p* value for the model mismatch is not significant (>.05), indicating other factors have little effect on the model. Therefore, it was concluded that the model can be used to analyze and predict the dependent variables correctly.

**Table 3:** The variance analysis for the solubility of gluten–maltose conjugates

Source	Square sum	Freedom	Mean square	*F* value	*p* value	Significance
Model	2.5963	9	0.2885	344.8858	<.0001	**
X_1_	0.1711	1	0.1711	204.5751	<.0001	**
X_2_	0.0242	1	0.0242	28.9325	.001	**
X_3_	0.0325	1	0.0325	38.8706	.0004	**
X_1_X_2_	0.0072	1	0.0072	8.6379	.0217	*
X_1_X_3_	0.0121	1	0.0121	14.4663	.0067	**
X_2_X_3_	0.0110	1	0.0110	13.1810	.0084	**
X_1_^2^	1.0802	1	1.0802	1,291.4168	<.0001	**
X_2_^2^	0.4420	1	0.4420	528.4423	<.0001	**
X_3_^2^	0.5811	1	0.5811	694.7446	<.0001	**
Residual	0.0059	7	0.0008			
Lack of fit	0.0046	3	0.0015	4.7656	.0828	
Pure Error	0.0013	4	0.0003			
The sum	2.6021	16				

* Significant, .01 ≤ *p* < .05.

** Very significant, *p* < .01; not significant, *p* ≥ .05.

#### 3.1.2 Response surface analysis

**FIGURE 1:**
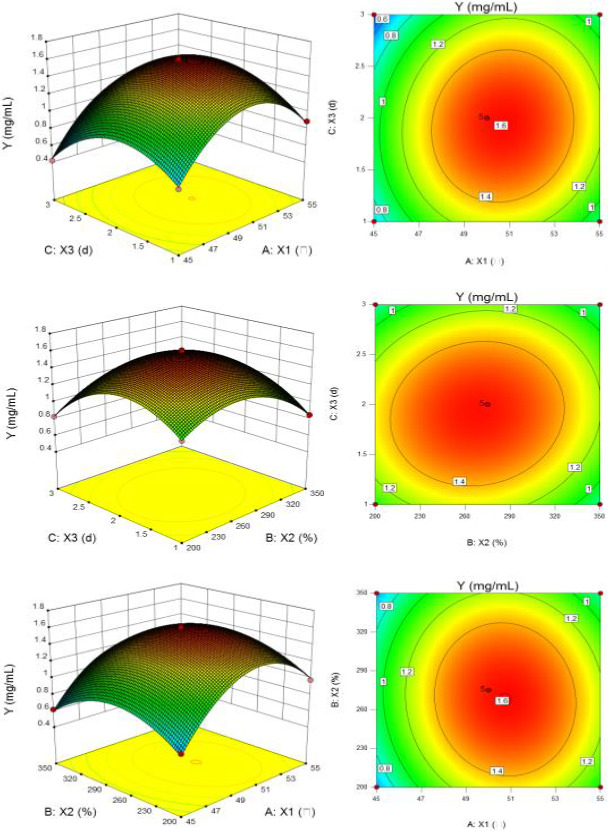
Two‐factor interactive response surface analysis for the solubility of gluten–maltose conjugates

With solubility as the dependent variable, the 3 days response surface was drawn (Figure 1). Figure 1 shows the effects of reaction temperature, reaction time, and gluten/maltose (W/W) on the solubility are bidirectional. When the reaction conditions in the central region, the solubility of gluten–maltose conjugates is the largest. The contour map shows that interaction between reaction temperature and gluten/maltose (W/ W) is significant. And the interaction between reaction temperature and reaction time, reaction time, and gluten/maltose (W/W) is very significant. The beneficial reaction conditions for improving the solubility are as follows: the reaction temperature range: 48~54°C; the gluten/maltose (W/W) range: 210%~320%; and the reaction time range: 1 ~ 2.5 days. The priority order of the influence factors on the solubility was reaction temperature > reaction time > gluten/maltose (W/W).

#### 3.1.3 Optimization and verification of the model

The optimal conditions for Maillard reaction of maltose suggested by the model to enhance the solubility were as follows: reaction temperature, 50.72°C; gluten/maltose ratio (W/W), 267.36%; and reaction time, 1.92 days. Under these conditions, the solubility is 1.60 mg/ml. In order to test the model, considering the actual conditions, the reaction conditions can be set to: the reaction temperature, 51°C; gluten/maltose ratio (W/W) 270%; and the reaction time, 1.92 days (46 hr). According to the optimized conditions, three sets of parallel experiments were done. The value was shown in Table 4. It can be seen from Table 4 that the experimental value is slightly different from the predicted value. However, they are not significantly different. The analysis results clearly show that RSM is sufficient for the optimization of Maillard reactions.

**Table 4:** The test verification table for the solubility of gluten–maltose conjugates

Types of sugar	Actual value/(mg/ml)			Mean value/(mg/ml)	Predictive value/(mg/ml)	Error/%
	Test 1	Test 2	Test 3			
Maltose	1.59	1.65	1.59	1.61	1.60	0.63

### 3.2 FTIR analysis of gluten–maltose conjugates

**FIGURE 2:**
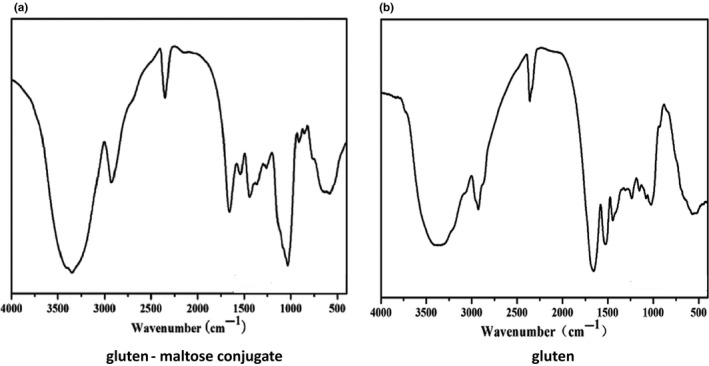
Infrared spectrum figure for gluten and gluten–maltose conjugate

**FIGURE 3:**
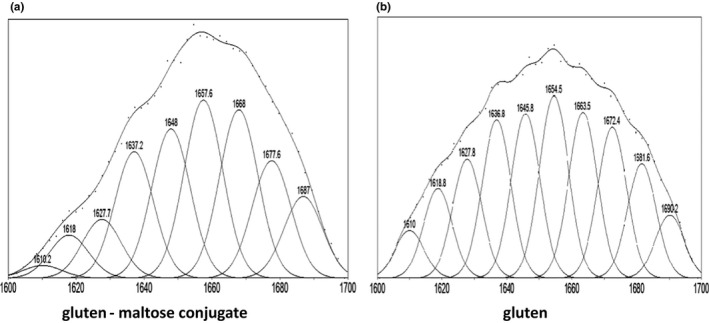
The infrared fitting curve of amide I band for gluten and gluten–maltose conjugate

FTIR spectroscopy is a particularly useful technique for studying protein–carbohydrate structures because there are several characteristic absorption bands in the infrared region, including Amide I (1600–1700 cm^−1^), Amide II (1400–1800 cm^−1^), and Amide III (1220–1330 cm^−1^) (Liu, Ru, & Ding, 2012; Yuan, Yue, Gao, & Sun, 2015). And Amide I (1600–1700 cm^−1^) can reflect the changes of secondary structure of glycosylated and unglycosylated proteins. This secondary structure is mainly composed of bed sheets (1600–1640 cm^−1^), bed helices (1650–1670 cm^−1^), and bed turns (1680–1685 cm^−1^). The bands at 1640–1650 cm^−1^ belong to the side chain structure (Yaping et al., 2014; Liu et al., 2012). Figure 2 shows the FTIR spectra of conjugate and gluten. The spectral was processed by the software Peak fit to carry out the convolution, the peak, and the fitting processing and to get the infrared fitting curve of the amide I band (Figure 3). Then, the peak area is calculated to get the proportion of different secondary structure (Table 5). Table 5 shows the proportion of α‐helices in the conjugate increased significantly, and β‐sheets had a larger decline. But the proportion of β‐turns and random coil had little change. In theory during the heating process, the protein peptide chain with thermal shock, the secondary bonds keeping the spatial structure of the protein were destroyed, and the molecular order arrangement of molecules was released with the expansion of the molecular structure of the protein, leading to the increase of the disorder structure of the protein. But the Maillard reaction conditions are mild with low temperature, and the protein peptide did not suffer excessive heat shock. So the increase of random coil was not obvious.

**Table 5:** The secondary structure of gluten and gluten–maltose conjugate

Structure type	Proportion of the second structure/%	
	Gluten	Gluten–maltose conjugate
b‐sheet	37.94	25.73
Random coil	14.27	15.94
a‐helix	25.7	37.05
b‐turn	22.09	21.28

In addition to the characteristic absorption bands of protein, each polystate has a specific hydrogen bond network involving OH, C = O, and NH groups, which leads to the complexity of the infrared spectrum (Pearce & Kinsella, 1978). When proteins and sugar molecules are bound by covalent bonds, an increase in the content of hydroxyl groups is a typical characteristic and shows a characteristic increase in the absorption of hydroxyl groups. Free hydroxyl has characteristic absorption peaks in the 3700–3200 cm^−1^, while the stretching vibration of polarity C = O bond had strong absorption peak in the 1200–1000 cm^−1^ (Yaping et al., 2014). As shown in Figure 2, the conjugate had stronger absorption peak in 3700–3200 cm^−1^ and 1200–1000 cm^−1^ compared to gluten, which confirmed sugar molecules accessed protein molecules by covalent bonding.

### 3.3 *SEM* analysis of gluten–maltose conjugates

**FIGURE 4:**
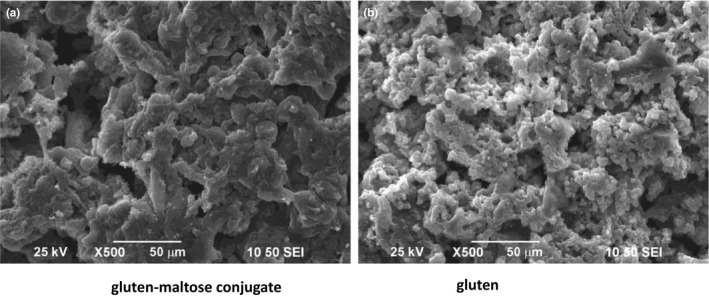
The scanning electron microscope figure for gluten and gluten–maltose conjugate

The scanning electron micrographs (*SEM*) of gluten and gluten–maltose conjugate, taken at 500× magnification, respectively, are shown in the Figure 4. It reveals what happens when maltose and proteins are dry‐heated under controlled conditions. The electron micrograph of the untreated gluten showed that the surface of the original gluten particles was massive, and the volume of the massive tissue was small. But the massive tissue of conjugates prepared by dry‐heating reaction under controlled conditions increased significantly. This proved that the gluten is strongly linked to the maltose making their conjugates. It looks very clear that gluten protein molecules are intimately associated with maltose molecules giving their closely associated compact and nonhomogeneous microstructure, which further confirms a covalent linkage between them.

## 4 CONCLUSIONS

The effects of reaction temperature, time, and gluten/maltose ratio (W/W) on the solubility of gluten–maltose conjugate were studied. And the reaction conditions were optimized by using Box‐Behnken model. The analysis of variance showed that the model was fit well with the experimental data and the experimental error was small. By the verification of the model, the best reaction conditions were as follows: The temperature was 50.72°C, the gluten/maltose ratio W/W was 267.36%, and the reaction time was 1.92 days. And the solubility was 1.60 mg/ml under the optimized reaction condition. The grafting reaction was proven by FTIR and *SEM*. FTIR also demonstrated that the incoming sugar chain made the protein spatial structure change, resulting in the change of the solubility of gluten. Scanning electron microscopy showed the microstructure of gluten–maltose conjugate and gluten is obviously different. The volume of the massive tissue of gluten was small, but the massive tissue of gluten–maltose conjugate increased significantly, proving that sugar binds to protein. In this work gluten–maltose conjugates showed better solubility than native gluten. Therefore, grafting reaction between gluten and polysaccharide is an efficient way to develop new use of gluten in food. Further investigations on physicochemical properties and functional characteristics of gluten–polysaccharide conjugates will be conducted, such as TGA analysis, DSC analysis, emulsifying activity and emulsifying stability analysis, and foaming property and foaming stability analysis, to achieve more improvements in protein glycosylation and elucidate the grafting mechanism.

## CONFLICT OF INTEREST

The authors declare no conflict of interest.

## ETHICAL APPROVAL

This study does not involve any human or animal testing.

## INFORMED CONTENT

Written informed consent was obtained from all study participants.
